# High Child-Pugh and CRUB65 scores predict mortality of decompensated cirrhosis patients with COVID-19: A 23-center, retrospective study

**DOI:** 10.1080/21505594.2021.1909894

**Published:** 2021-04-19

**Authors:** Yong Xiao, Dongwen Wu, Xiao Shi, Shuzhong Liu, Xudong Hu, Chenliang Zhou, Xia Tian, Huimin Liu, Hui Long, Zhihong Li, Ji Wang, Tao Tan, Ying Xu, Bitao Chen, Ting Liu, Heng Zhang, Shihua Zheng, Shunlin Hu, Jun Song, Jie Tang, Jichun Song, Zhengwei Cheng, Weitian Xu, Yongxiang Shen, Wenhu Yu, Yong Xu, Jiao Li, Jing Zhou, Fen Wang, Mingkai Chen

**Affiliations:** a Department of Gastroenterology, Renmin Hospital of Wuhan University, Wuhan, Hubei, China; b Department of Gastroenterology, The Third XiangYa Hospital Central South University, Changsha, Hunan, China; c Department of Gastroenterology, Wuhan Jinyintan Hospital, Wuhan, Hubei, China; d Intensive Care Unit, Renmin Hospital of Wuhan University, Wuhan, Hubei, China; e Department of Gastroenterology, Tongren Hospital of Wuhan University (Wuhan Third Hospital), Wuhan, Hubei, China; f Department of Gastroenterology, The Second Affiliated Hospital of Jianghan University (Wuhan Fifth Hospital), Wuhan, Hubei, China; g Department of Gastroenterology, Tianyou Hospital, Wuhan University of Science and Technology, Wuhan, Hubei, China; h Emergency Center, the Central Hospital of Xiaogan, Xiaogan, Hubei, China; i Department of Gastroenterology, Hanyang Hospital Affiliated to Wuhan University of Science and Technology, Wuhan, Hubei, China; j Department of Gastroenterology, Third People’s Hospital of Hubei Provincial, Wuhan, Hubei, China; k Department of Gastroenterology, Wuhan Hankou Hospital, Wuhan, Hubei, China; l Department of Gastroenterology, Jingmen No.1 People’s Hospital, Jingmen, Hubei, China; m Department of Gastroenterology, Wuhan Ninth Hospital, Wuhan, Hubei, China; n Department of Gastroenterology, the Central Hospital of Wuhan, Tongji Medical College, Huazhong University of Science and Technology, Wuhan, Hubei, China; o Department of Gastroenterology, Yichang Central People’s Hospital, Yichang, Hubei, China; p Department of Gastroenterology, Xiangyang No.1 People’s Hospita, Xiangyang, Hubei, China; q Department of Gastroenterology, Union Hospital Affiliated to Huazhong University of Science and Technology, Wuhan, Hubei, China; r Department of Orthopedic, Wuhan Fourth Hospital, Wuhan, Hubei, China; s Department of Gastroenterology, Chibi Genral Hospital, Chibi, Hubei, China; t Department of Gastroenterology, Tongji Xianning Hospital, Huazhong University of Science and Technology, Xianning, Hubei, China; u Department of Gastroenterology, Central Theater General Hospital, Wuhan, Hubei, China; v Department of Gastroenterology, The First People’s Hospital of Tianmen Hubei Province, Tianmen, Hubei, China; w Department of Gastroenterology, Xiantao First People’s Hospital, Xiantao, China; x Department of Gastroenterology, Tongcheng People’s Hospital, Tongcheng, Hubei, China

**Keywords:** COVID-19, decompensated cirrhosis, risk factor

## Abstract

**Background**: COVID-19 has rapidly become a major health emergency worldwide. The characteristic, outcome, and risk factor of COVID-19 in patients with decompensated cirrhosis remain unclear.

**Methods**: Medical records were collected from 23 Chinese hospitals. Patients with decompensated cirrhosis and age- and sex-matched non-liver disease patients were enrolled with 1:4 ratio using stratified sampling.

**Results**: There were more comorbidities with higher Chalson Complication Index (p < 0.001), higher proportion of patients having gastrointestinal bleeding, jaundice, ascites, and diarrhea among those patients (p < 0.05) and in decompensated cirrhosis patients. Mortality (p < 0.05) and the proportion of severe ill (p < 0.001) were significantly high among those patients. Patients in severe ill subgroup had higher mortality (p < 0.001), MELD, and CRUB65 score but lower lymphocytes count. Besides, this subgroup had larger proportion of patients with abnormal (PT), activated partial thromboplatin time (APTT), D-Dimer, alanine aminotransferase (ALT), aspartate aminotransferase (AST), total bilirubin (TBL) and Creatinine (Cr) (p < 0.05). Multivariate logistic regression for severity shown that MELD and CRUB65 score reached significance. Higher Child-Pugh and CRUB65 scores were found among non-survival cases and multivariate logistic regression further inferred risk factors for adverse outcome. Receiver Operating Characteristic (ROC) curves also provided remarkable demonstrations for the predictive ability of Child-Pugh and CRUB65 scores.

**Conclusions**: COVID-19 patients with cirrhosis had larger proportion of more severely disease and higher mortality. MELD and CRUB65 score at hospital admission may predict COVID-19 severity while Child-Pugh and CRUB65 score were highly associated with non-survival among those patients.

## Introduction

Decompensated cirrhosis is end-stage of chronic liver disease with microbial infection acting as its major complication and cause of the death [1,2]. Boivin Z et al. suggested that physicians should monitor cirrhotic patients carefully to prevent 90-day mortality caused by pneumonia [3]. A recent Coronavirus Disease 2019 (COVID-19) study with 21 cirrhosis patients (compensated vs. decompensated: 17 vs. 4) shown that lymphocyte, platelets count, and bilirubin level were associated with their high mortality (23.8%) [4]. However, there have been few studies focusing on clinical features and risk factors of adverse outcome in COVID-19 patients with decompensated cirrhotic. This study aimed to describe characteristics, as well as to identify risk factors associated with severity and adverse outcomes of those patients.

## Method

### Study design and participants

Among 15,732 COVID-19 patients from 23 medical centers in central China between January and March 2020, 66 decompensated cirrhotic patients were identified (Supplemental Table 1). We enrolled age and sex-matched 264 patients without liver diseases at the ratio 1:4 from corresponding hospitals to reduce statistical linkage on bias by stratified sampling.

### Data collection

Electronic medical records were collected, which included: demographic characteristics, disease comorbidities, clinical manifestations (COVID-19 related symptoms: fever, cough, expectoration, myalgia, headache, dyspnea, etc.; cirrhosis related symptoms: black stool, hematemesis, abdominal distention, hydrothorax, etc.), laboratory examination (complete blood counts: white blood cells, lymphocytes, red blood cell, hemoglobin and platelets (PLT); serum biochemistry test: alanine aminotransferase (ALT), aspartate aminotransferase (AST), albumin (ALB) and creatinine (Cr); coagulation profile), therapy (antiviral, antimicrobial, corticosteroid, respiratory support, immunoglobulin, plasma products, etc.) and treatment outcomes (discharge, hospitalization or death). Three researchers reviewed data independently. For patients whose epidemiological or symptom data could not be obtained from records, themselves or their family members will be contacted.

### Definition

The diagnosis criteria of COVID-19 and the disease severity classification were based on the Chinese management guideline for COVID-19 (version 6.0) established by the National Health Commission of the People’s Republic of China [5]. Patients who met any of the following criteria were assigned into “Severe” subgroup: 1) shortness of breath, respiratory rate >30 breath per minute (bpm); 2) oxygen saturation ≤93%; 3) Arterial oxygen partial pressure (PaO2)/fraction of inspired oxygen (FiO2) ≤ 300 mmHg; 4) lung imaging shows a substantial progression of lesions (greater than 50%) within 24–48 h. The diagnosis criteria of decompensated cirrhosis were based on the European Association for the Study of the Liver (EASL) clinical practice guidelines for the management of patients with decompensated cirrhosis and Chinese guidelines on the management of liver cirrhosis [6,7].

### Ethics

Approval for this study was obtained from the Ethics Committee of Renmin Hospital of Wuhan University (reference number: WDRY2020-K171). A waiver for informed consent was also approved due to the retrospective nature of this study and analysis was carried out anonymously.

### Statistical analysis

Categorical variables were described as frequency and percentage, while continuous variables were illustrated by average, median, and quartile spacing (IQR). Student’s t-test would be applied to compare the mean of continuous variables if normally distributed; otherwise, Mann–Whitney U test would be utilized. Categorical variables were analyzed by chi-square tests or Fisher’s exact test. Univariate and multivariate logistic regression was used to explore risk factors that could predict the severity and outcomes. As we enrolled 38 patients in the severe subgroup and 11 non-survival cases, only 5 factors for severity and 2 factors for survival were tested in multivariate logistic regression in case of overfitting. We considered a P value less than 0.05 as statistically significant. Discrimination abilities of scores were assessed with Receiver Operating Characteristic (ROC) curves. The Area Under the Curve (AUC) was calculated and the maximum Youden’s Index (J = sensitivity + specificity − 1) was employed to define an optimal cutoff point. Statistical analyses were performed in SPSS software version 16.0 for Windows (SPSS, Inc.) and Medcalc software version 19.7.

## Results

### Patient characteristics

**Figure 1.**
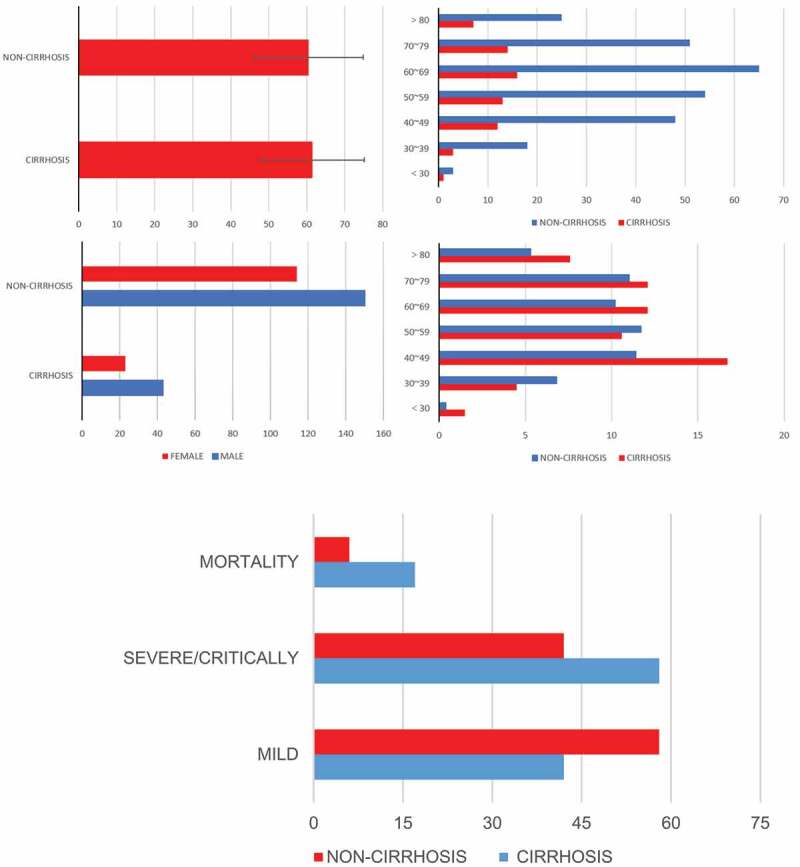
Age and gender distribution of patients

Decompensated cirrhosis group included 43 men and 23 women with an average age of 61.5 ± 13.6 years, while the non-liver disease group enrolled 150 men and 114 women with an average age of 60.5 ± 14.4 (Figure 1).

### COVID-19 patients with decompensated cirrhosis vs. patients without liver disease

The most common COVID-19 symptoms in decompensated cirrhosis group are shortness of breath (56.1%), fever (43.9), and cough (40.9%), while fever (83.7%), cough (75%), and fatigue (43.9) were more common in patients without liver disease. Among patients who had a fever, decompensated cirrhosis group had a higher proportion of high fever (p < 0.05). And those patients had more gastrointestinal (GI) symptoms at hospital admission, such as GI bleeding, ascites, jaundice, and diarrhea (p < 0.05). Besides, more comorbidities, including hypertension (28.8% vs 14.4%, p = 0.006), diabetes (15.2% vs 6.4%, p = 0.02),and respiratory system diseases (COPD, tuberculosis et al, 13.6% vs 5.3%, p = 0.04) and CCI (0.83 ± 1.26 vs 0.08 ± 0.47, p < 0.001) were found in those patients, but there was no difference in cardiovascular disease, urinary system disease, and neoplasia (Table 1).

**Table 1.** Characteristics of COVID-19 patients

Variable	Patients with decompensated cirrhosis (n = 66)	Patients without liver disease (n = 264)	P value
**Comorbidities**			
Hypertension	19 (28.8%)	38 (14.4%)	0.006
Cardiovascular disease	5 (7.6%)	14 (5.3%)	0.68
Diabetes	10 (15.2%)	17 (6.4%)	0.02
Respiratory	9 (13.6%)	14 (5.3%)	0.04
Renal system	4 (6.1%)	10 (3.8%)	0.63
Malignancy	5 (7.6%)	5 (1.9%)	0.031
Charlson Comorbidity Index	0.83 ± 1.26	0.08 ± 0.47	<0.001
**Chief complaints on admission**			
Hemorrhage	6 (9.1%)	2 (0.8%)	<0.001
Ascites	25 (38%)	0 (0%)	<0.001
icterus admission	16 (24.2%)	14 (5.3%)	<0.001
Cough	27 (40.9%)	198 (75%)	<0.001
Myalgia or fatigue	28 (42.4%)	116 (43.9%)	0.82
Headache	4 (6%)	20 (7.6%)	0.796
Hemoptysis	2 (3%)	4 (1.5%)	0.345
Diarrhea	9 (13.6%)	15 (5.7%)	0.05
Polypnea	37 (56.1%)	110 (41.7%)	0.04
Fever	29 (43.9%)	221 (83.7%)	<0.001
**Highest temperature**, °C			
37.3–38	9 (31.0%)	111 (50.2%)	-
38–39	8 (27.9%)	71 (32.1%%)	-
>39	12 (41.4)	38 (17.2%)	
**Blood Routine**			
White blood cell count, 10⁹/L	5.89 ± 0.53	6.05 ± 0.18	0.77
<4	25 (37.9%)	60 (22.7%)	0.012
4–10	29 (43.9%)	183 (69.3%)	<0.001
>10	12 (18.2%)	21 (8.0%)	0.013
Neutrophil count, 10⁹/L	4.59 ± 0.49	4.70 ± 0.38	0.89
Lymphocyte count, 10⁹/L	0.79 ± 0.07	1.10 ± 0.46	0.003
<1.0	48 (72.7%)	129 (48.9%)	0.001
Red blood cell count, 1012/L	3.66 ± 0.89	4.23 ± 0.6	<0.001
Hemoglobin, g/L	110.1 ± 26.5	129.6 ± 17.9	<0.001
PLT	114.8 ± 11.5	201.5 ± 15.06	<0.001
**Coagulation function**			
Prothrombin time, sec	15.9 ± 5.6	13.7 ± 5.5	0.005
Activated partial thromboplastin, sec	35.1 ± 10.1	31.7 ± 7.9	0.02
D-dimer, mg/L	5.28 ± 1.8	1.72 ± 0.32	0.001
≥1	33 (86.8%)	57 (21.6%)	<0.001
**Liver function**			
ALB, g/L	31.4 ± 6.7	36.8 ± 5.4	<0.001
<27	17 (25.8%)	18 (6.8%)	<0.001
27–30	13 (19.7%)	34 (12.9%)	0.16
≥30	36 (54.5%)	212 (80.3%)	<0.001
ALT, U/L	68.84 ± 18.01	35.16 ± 2.49	0.069
<40	39 (59.1%)	196 (74.2%)	0.015
40–100	15 (22.7%)	54 (20.5%)	0.69
≥100	12 (18.2%)	14 (5.3%)	0.001
AST, U/L	90.72 ± 22.57	37.92 ± 3.5	0.024
<40	23 (34.8%)	217 (82.2%)	<0.001
40–100	33 (50%)	37 (14%)	<0.001
≥100	10 (15.2%)	10 (3.8%)	0.001
TBIL, μmol/L	42.8 ± 8.72	12.65 ± 0.56	0.001
**Kidney function**			
Creatinine, μmol/L	117.03 ± 15.89	114.96 ± 10.68	0.93
≥133	12 (18.2%)	38 (14.4%)	0.44
**Treatments**			
Antibiotics	66 (100%)	264 (100%)	NA
Antiviral treatment	66 (100%)	264 (100%)	NA
Corticosteroids	19 (29%)	50 (18.9%)	0.078
Intravenous immunoglobin	18 (27.3%)	79 (29.9%)	0.672
High-flow nasal cannula	34 (51.5%)	187 (70.8%)	0.003
Noninvasive mechanical ventilation	8 (12.1%)	28 (10.6%)	0.724
Invasive mechanical ventilation/ECMO	5 (7.6%)	12 (4.5%)	0.349

Complete blood count showed COIVD-19 patients with decompensated cirrhosis had lower RBC and platelets count (p < 0.001), less hemoglobin (p < 0.001) but a higher proportion of patients with abnormal WBC (<4*109/L, >10*109/L) and lymphocytes (<1*10^9^/L) count. Prolonged prothrombin time (PT), activated partial thromboplastin time (APTT), and evaluated D-Dimer, as well as a higher proportion of patients having abnormal D-Dimer (>1 mg/ml) were found in those patients’ coagulation profile (p < 0.05). What’s more, in serum biochemical tests (including renal and liver function), they showed elevated ALT, AST, and TBIL, but reduced ALB. The proportion of patients with abnormal ALB (<27 g/L), ALT (>100 U/L) and AST (>100 U/L) were also significantly high in decompensated cirrhotic COVID-19 patients (p < 0.001), while Cr in serum shown no difference (Table 2).

**Table 2.** Characteristics of decompensated cirrhosis patients with COVID-19

Variable	Severe (n = 38)	Nonsevere (n = 28)	P value
Age (years, IQR)	60.2 ± 10.9	63.4 ± 16.9	0.39
Male	26 (68.4%)	14 (60.7%)	0.52
**Etiology of cirrhosis**			0.80
Hepatitis	25 (65.8%)	16 (57.1%)	0.47
Alcohol	2 (5.3%)	3 (10.7%)	0.64
Schistosome	4 (10.5%)	4 (14.3%)	0.71
Others	7 (18.4%)	5 (17.9%)	0.95
Splenectomy	5 (13.2%)	1 (3.6%)	0.23
**Child-Pugh Class**	8.68 ± 3.06	6.54 ± 1.93	0.001
A	12 (31.6%)	20 (71.4%)	0.01
B	13 (34.2%)	5 (17.9%)	0.14
C	13 (34.2%)	3 (10.7%)	0.03
**MELD**	19.1 ± 9.66	10.5 ± 3.6	<0.001
<10	6 (15.8%)	14 (50%)	0.003
10 ~ 20	15 (39.5%)	13 (46.4%)	0.57
≥20	17 (44.7%)	1 (3.6%)	<0.001
**CURB-65 SCORE**	2.82 ± 0.77	1.39 ± 0.5	<0.001
0–1	3 (7.9%)	15 (53.6%)	<0.001
2	12 (31.6%)	11 (39.3%)	0.52
3–5	23 (60.5%)	2 (7.1%)	<0.001
**Comorbidities**			
No comorbidities	10 (26.3%)	5 (17.9%)	0.42
Hypertension	12 (31.6%)	7 (25%)	0.56
Cardiovascular disease	3 (7.9%)	2 (7.1%)	0.91
Diabetes	8 (21.1%)	2 (7.1%)	0.12
Chronic pulmonary disease	4 (10.5%)	5 (17.9%)	0.62
Chronic renal insufficiency	4 (10.5%)	0 (0%)	0.13
Charlson Comorbidity Index	1.03 ± 1.48	0.57 ± 0.82	0.153
**Signs and symptoms**			
Hemorrhage	3 (7.9%)	3 (10.7%)	0.69
Ascites	21 (55.3%)	4 (14.3%)	0.001
Icterus admission	13 (34.2%)	3 (10.7)	0.03
Cough	19 (50%)	8 (28.6%)	0.08
Myalgia or fatigue	16 (42.1%)	12 (42.9%)	0.95
Headache	3 (7.9%)	1 (3.6%)	0.63
Hemoptysis	1 (2.6%)	1 (3.6%)	0.83
Diarrhea	6 (15.8%)	3 (10.7%)	0.82
Fever	18 (47.4%)	11 (39.3%)	0.51
**Highest temperature**, °C			
37.3–38	3 (7.9%)	6 (21.4%)	0.22
38–39	4 (10.5%)	4 (14.3%)	0.71
>39	11 (28.9%)	1 (3.6%)	0.008
**Blood Routine**			
White blood cell count, 10⁹/L	6.61 ± 4.85	5.01 ± 3.08	0.13
<4	12 (31.6%)	13 (46.4%)	0.22
4–10	17 (44.7%)	12 (42.9%)	0.88
>10	9 (23.7%)	3 (10.7%)	0.18
Neutrophil count, 10⁹/L	5.45 ± 4.52	3.5 ± 2.7	0.49
Lymphocyte count, 10⁹/L	0.60 ± 0.4	1.04 ± 0.6	0.002
<1.0	33 (86.8%)	15 (53.6%)	0.003
Red blood cell count, 1012/L	5.46 ± 4.52	3.50 ± 2.73	0.49
Hemoglobin, g/L	104.4 ± 25.5	117 ± 26.5	0.06
PLT	101 ± 17.0	132 ± 14.2	0.19
**Coagulation function**			
Prothrombin time, sec	17.7 ± 6.7	13.6 ± 2.1	0.002
Activated partial thromboplastin, sec	36.9 ± 11	32.7 ± 8.6	0.013
D-dimer, mg/L	6.2 ± 2.4	2.6 ± 1.2	0.38
≥1	25 (65.8%)	8 (28.6%)	0.003
**Liver function**			
ALB, g/L	29.2 ± 6.1	34.2 ± 6.6	0.003
<27	13 (34.2%	4 (14.3%)	0.09
27–30	7 (18.4%)	6 (21.4%)	0.35
≥30	18(47.4%)	18 (64.3%)	0.17
ALT, U/L	91.73 ± 31.4	39.4 ± 5.5	0.11
<40	20 (52.6%)	19 (67.9%)	0.21
40–100	8 (21.1%)	7 (25%)	0.71
≥100	10 (26.3%)	2 (7.1%)	0.046
AST, U/L	125.2 ± 39.3	46.4 ± 14.6	0.054
<40	16 (42.1%)	7 (25%)	0.15
40–100	13 (34.2%)	20 (71.4%)	0.003
≥100	9 (23.7%)	1 (3.6%)	0.02
TBIL, μmol/L	62.5 ± 15.2	19.4 ± 10.1	0.008
**Kidney function**			
Creatinine, μmol/L	130.7 ± 22.8	99.9 ± 21.6	0.34
≥133	10 (26.3%)	2 (7.1%)	0.046
**Death**	11 (28.9%)	0 (0%)	<0.001

COIVD-19 patients with decompensated cirrhosis had more severe/critically ill cases (57.5% vs 42%, p < 0.01) and higher mortality (17% vs 6%, p < 0.001) (Figure 1). As for therapy, patients without the liver disease had high proportion of patients using high flow nasal cannula (p = 0.003), but there was no difference in antibiotics, antivirus, glucocorticoid, γ globulin, invasive/noninvasive ventilator and ECMO using (Table 1).

### Comparison between severe and non-severe subgroup among COVID-19 patients with decompensated cirrhosis

Death cases in COIVD-19 patients with decompensated cirrhosis were all from severe ill subgroup (28.9% vs 0%). Significant difference was also found in patients percentage of Child-Pugh C (34.2% vs 10.7%, P = 0.03), high MELD score (≥20,44.7% vs 3.6%, P < 0.001), ascites (55.3% vs 14.3%, P = 0.001), jaundice (34.2% vs 10.7%, P = 0.03) and high fever (>39°C, 28.9% vs 3.6%, P = 0.008) between severe and non-severe subgroup (Table 3). However, there was no difference in age, gender, etiology of cirrhosis, underlying condition, history of splenectomy, GI bleeding, cough, hemoptysis, muscle pain, fatigue, and diarrhea.

**Table 3.** Risk factors for severe/critical COVID-19 in decompensated cirrhosis patients

Variable	Univariable OR (95%CI)	P value	Multivariable OR (95%CI)	P Value
**Age**	1.01 (0.97–1.05)	0.62	.	.
Age (<40)	0.23 (0.02–2.29)	0.21	.	.
40–50	1.04 (0.29–3.69)	0.95	.	.
50–60	1.23 (0.35–4.25)	0.75	.	.
Age (>60)	1.33 (0.50–3.57)	0.57	.	.
Age (>65)	1.2 (0.45–3.21)	0.72	.	.
**Gender**	0.54 (0.20–1.52)	0.24	.	.
**Charlson Comorbidity Index**	0.71(0.44–1.16)	0.17		
**Child-Pugh SCORE**	1.40 (1.11–1.77)	0.005	1.18 (0.82–1.69)	0.380
A	0.14 (0.05–0.43)	<0.001	.	.
B	3 (0.94–9.62)	0.07	.	.
C	4.33 (1.1–17.09)	0.04	.	.
ALB	0.87 (0.79–0.96)	0.004	.	.
TBIL	1.04 (1–1.08)	0.04	.	.
PT	1.42 (1.13–1.80)	0.003	.	.
**MELD SCORE**	1.25 (1.10–1.43)	<0.001	1.30 (1.06–1.60)	0.011
<10	0.29 (0.09–0.92)	0.04	.	.
10 ~ 20	0.61 (0.23–1.63)	0.32	.	.
≥20	17.61 (2.16–143.69)	0.007	.	.
**Creatine U/L**	1.00 (0.99–1.01)	0.34	.	.
**D-dimer, g/L**	1.17 (0.97–1.41)	0.11	1.05 (0.93–1.19)	0.41
<1	0.56 (0.19–1.64)	0.29	.	.
≥1	1.79 (0.61–5.25)	0.29	.	.
**Lymphocyte count, 10⁹/L**	0.16 (0.05–0.533)	0.003	0.93 (0.19–4.48)	0.93
<1.0	5.7 (1.73–18.96)	0.004	.	.
≥1	0.18 (0.05–0.58)	0.004	.	.
**CURB65 SCORE**	11.47 (3.59–36.6)	<0.001	11.58 (2.18–61.36)	0.004
0–1	0.02 (0.003–0.195)	0.001	.	.
2	0.71 (0.26–1.98)	0.52	.	.
3–5	25 (5.12–122.19)	<0.001	.	

In laboratory examination, significant prolonged PT (17.7 vs 13.6 sec, P = 0.013), lower ALB (29.2 vs 34.2 g/L, P = 0.003) but higher TBIL (62.5 VS 19.4 μmol/L, p = 0.008) as well as higher proportion of patients who had abnormal lymphocytes count (<1 × 10⁹/L, 86.8% vs 53.6%, P = 0.002), D-Dimer (≥1 mg/L, 65.8% vs 28.6%, P = 0.003), ALT (>100 U/L, 26.3% vs 7.1%, p = 0.046), AST (>100 U/L, 23.7% vs 3.6%, p = 0.02) and Cr (>133 μmol/L, 26.3% vs 7.1%, P = 0.046) was found in severe ill subgroup.

### Risk factors of COVID-19 severity among decompensated cirrhosis patients

CCI, Child-Pugh, MELD and CURB65 score, abnormal lymphocytes count (<1 × 10^9^/L) and D-Dimer level were included in multivariate logistic regression for COVID-19 severity (Table 4), which showed that MELD (odds ratio 1.3, 95%CI 1.06–1.60, p = 0.011) and CURB65 (odds ratio 11.58, 95%CI 2.18–61.36, p = 0.004) score were associated with COVID-19 severity.

**Table 4.** Risk factors for death in decompensated cirrhosis patients with COVID-19

Variable	Univariable analysis			Multivariable analysis	
	**Survived (n = 55)**	**Died (n = 11)**	**P value**	**OR (95%CI) for death**	**P Value**
**Age**	66.63 ± 10.49	60.49 ± 14.16	0.18	.	.
**Gender (male)**	8 (72.7%)	35 (63.6%)	0.57	.	.
**Charlson Comorbidity Index**	1.36 ± 1.67	0.73 ± 1.14	0.15		
**PLT**	111.36 ± 10.8	136.09 ± 40.4	0.41	.	.
**Child-Pugh SCORE**	-	-	0.012	5.71 (1.85–17.72)	0.003
A	1 (9.1%)	31 (56.4%)	.	.	.
B	5 (45.5%)	13 (23.6%)	.	.	.
C	5 (45.5%)	11 (20%)		.	.
ALB	32.02 ± 6.78	27.43 ± 5.24	0.04	.	.
TBIL	36.54 ± 7.78	84.37 ± 39.84	0.058	.	.
DBIL	20.4 ± 5.25	64.57 ± 29.25	0.04	.	.
PT	14.98 ± 3.73	21.22 ± 3.42	0.008	.	.
**MELD SCORE**	14.35 ± 8.5	20.18 ± 9.7	0.06	.	.
<10	19 (28.8%)	1 (9.1%)	0.16	.	.
10 ~ 20	22 (40%)	6 (54.5%)		.	.
≥20	14 (27.3%)	4 (36.4%)		.	.
Creatine U/L	105.33 ± 15.68	166.78 ± 49.48	0.06	.	.
**D-dimer, g/L**	3.8 ± 1.18	13.02 ± 2.68	0.08	.	.
≥1	24 (43.6%)	9 (81.8%)	0.033	.	.
**Lymphocyte count, 10⁹/L**	0.85 ± 0.08	0.44 ± 0.08	0.035	.	.
<1.0	37 (56.1%)	11 (100%)	0.99	.	.
**CURB65 SCORE**	2.11 ± 0.12	3.09 ± 0.21	0.005	5.88 (1.96–17.68)	0.002
0–1	18 (27.3%)	0 (0%)	0.007	.	.
2	21 (38.2%)	2 (18.2%)		.	.
3–5	16 (29.1%)	9 (81.8%)		.	.

### Risk factors of COVID-19 adverse outcome among decompensated cirrhosis patients

**Figure 2.**
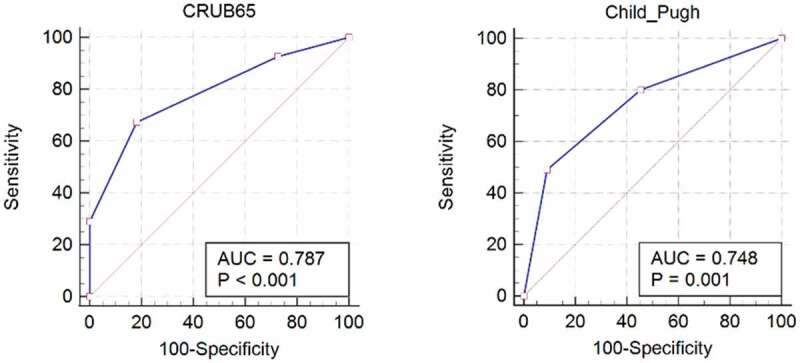
Receiver Operating Characteristic (ROC) curves analysis for CRUB65 and Child-Pugh scores including the area under the curve (AUC) and P-value

Differences in Child-Pugh, MELD and CURB65 score, DBIL and PT level, lymphocytes count, and patients proportion of abnormal D-Dimer (≥1 mg/L) reached significance between survival and non-survival cases, but not age, gender, PLT count, TBIL level, PT, Cr level, and MELD score. multivariate logistic regression showed that Child-Pugh (OR 5.71, 95%CI 1.85–17.72, p = 0.003) and CRUB65 (OR 5.88, 95%CI 1.96–17.68, p = 0.002) were highly associated non-survival (Table 4). In a ROC analysis for the mortality and survival, the area under ROC for CRUB65 was 0.787 (95% CI: 0.669–0.878), for Child-Pugh 0.748 (95% CI: 0.626–0.847), respectively (Figure 2). The optimum cutoff point for predicting mortality was 2 for the CRUB65 score (CRUB65 ≤ 2 for survival and CRUB65 ≥ 3 for mortality). At this value, the sensitivity was 67.27%, the specificity 81.82%, the positive predictive value 94.9% and the negative predictive value 33.3%. The optimum cutoff point for predicting mortality was 1 for the Child-Pugh score (Child-Pugh class A for survival and class B/C for mortality). At this value, the sensitivity was 49.09%, the specificity 90.91%, the positive predictive value 96.4% and the negative predictive value 26.3% (Table 5).

**Table 5.** Calculation of sensitivity, specificity, PPV and NPV at cutoffs for CRUB65 and Child-Pugh scores

Score	Cutoff	Sensitivity (95%)	Specificity (95%)	Positive predictive value (95%)	Negative predictive value (95%)
CRUB65	≤2	67.27% (53.3–79.3)	81.82% (48.2–97.7)	94.9% (83.9–98.5)	33.3% (23.8–44.5)
Child-Pugh	≤1	49.09% (35.4–62.9)	90.91% (58.7–99.8)	96.4% (80.3–99.4)	26.3% (20.6–33.0)

## Discussion

Our previous study suggested that the incidence of COVID-19 in decompensated cirrhotic patients from January 1 to February 4, 2020, in Wuhan, China was around 17% [8], and most of them were severely ill case. The purpose of this multi-center study was to further investigate the clinical characteristic and risk factors of adverse outcomes in decompensated cirrhotic patients with SARS-Cov-2 infection. Our results revealed that the proportion of severely ill and mortality in COIVD-19 patients with decompensated cirrhotic was significantly higher than patients without liver disease. Besides, high MLED and CRUB65 score at hospital admission were associated with COVID-19 severity, while Child-Pugh and CURB65 score was associated with survival.

Previous studies have shown that 14–53% of COVID-19 patients had elevated ALT and AST which was associated with disease severity [9,10]. Fan et al. demonstrated that patients with abnormal liver function had more cough cases but no difference in comorbidities (hypertension, diabetes, and respiratory system disease, etc.) [10]. But in our studies, we found COVID-19 patients with decompensated cirrhosis had more hypertension and diabetes as well as GI symptoms, including GI bleeding, ascites, jaundice, and diarrhea. This may be explained by that decompensated cirrhosis patients were relatively old and had impaired liver-gut axis [11,12].

Interestingly, a national study has found that patients with any comorbidity yielded poorer clinical outcomes than those without [13]. Our results showed that although the CCI was also significantly higher in decompensated COVID-19 patients than in the control group, there was no difference between severe patients and nonsevere patients. Meanwhile, regression analysis showed no significant causal relationship with the severity and clinical outcome of COVID-19. Multiple comorbidities are a clinical feature of patients with cirrhosis [14], but they may not be the primary cause of disease outcomes.

Several studies have shown that severely ill cases in COVID-19 were characterized as elderly, reduced lymphocyte count, evaluated D-Dimer, venous thromboembolism formation, and having multiple comorbidities [15–17]. In our study, we found that decompensated cirrhotic infected with SARS-CoV-2 had decreased lymphocytes and RBC count, lower hemoglobin, and ALB level, prolonged PT, and APTT as well as evaluated ALT, AST, and TBIL compared with COVID-19 patients without liver disease.

In our study, 57.5% of COVID-19 patients with decompensated cirrhotic were severely ill with high mortality (28.9%), which was much higher than patients without liver disease. Angiotensin-converting enzyme 2 (ACE2) is the target of SARS-CoV-2 [18], which is also expressed on hepatic cell [9,19,20]. A recent autopsy also confirmed that SARS-CoV-2 injured hepatic cells directly [10]. Except for direct effects, global stress and inflammation induced by SARS-CoV-2, liver ischemia-reperfusion injury, previous liver disease aggravation, and medical treatment all could cause liver injury [9,21]. Decompensated cirrhotic patients also had impaired immune response to infection [2,22]. Portal hypertension leads to the imbalance of intestinal flora and intestinal barrier dysfunction [23], cytokine storm, and disorders of portal hemodynamics, all of which could accelerate acute liver function failure [22]. All those mentioned factors contributed to a higher proportion of severely illness and mortality in those patients.

Previous studies suggested that age and D-Dimer were associated with the severity and outcome of COVID-19. However, in this study, we did not repeat that correlation among COVID-19 patients with decompensated cirrhosis, which may because they were elderly and had abnormal D-Dimer. Child-Pugh and MELD score are models to predict the prognosis of cirrhotic [24,25]. In patients admitted to ICU, MELD score had higher sensitivity than Child–Pugh score, but for chronic liver failure patients Child–Pugh score had higher sensitivity but lower specificity than MELD score [25]. A newly study enrolled 152 patients with liver disease (decompensated cirrhosis patients: 36.7%) shown that Child-Pugh and new hepatic decompensation events during COVID-19 were associated with survival [26]. Our results demonstrated that MELD score at hospital admission could be used for predicting COVID-19 severity, while Child-Pugh had it advantage to predict survival. CURB65 is a severity-predicting model for community-acquired pneumonia [27]. We proved that it could be used to predict not only severity but also survival of COVID-19 patients with decompensated cirrhosis.

Still, our study had several limitations. Firstly, no portal hemodynamic-related readouts (hepatic venous pressure gradient, a ratio of portal/splenic vein diameter, and thrombus formation) were analyzed, which may influence patients’ prognosis. Secondly, long-term outcomes of SARS-CoV-2 infection were not explored in those patients. Thirdly, there may be systematic bias due to the retrospective study method. Additional larger sample size RCT study are still in great need.

To our knowledge, this is the first study exploring the characteristics and risk factors of severity and survival for COIVD-19 patients with decompensated cirrhotic. Briefly, we found that those patients had a high proportion of severe/critically ill and mortality. MLED and CRUB65 score at hospital admission was associated with COVID-19 severity, while Child-Pugh and CURB65 could be used to predict patients’ survival.

## Supplementary Material

Supplemental Material

## References

[1] Bajaj JS, O’Leary JR, Reddy KR, et al. Second infections independently increase mortality in hospitalized patients with cirrhosis: the North American consortium for the study of end‐stage liver disease (NACSELD) experience. Hepatology. 2012;56(6):2328–2335. 22806618 10.1002/hep.25947 PMC3492528

[2] Piano S, Singh V, Caraceni P, et al. Epidemiology and effects of bacterial infections in patients with cirrhosis worldwide. Gastroentology. 2019;156(5):1368–1380.e10. 10.1053/j.gastro.2018.12.005 30552895

[3] Boivin Z, Perez MF, Atuegwu NC, et al. Impact of cirrhosis on pneumonia-related outcomes in hospitalized older veterans. Am J Med Sci. 2019;357(4):296–301. 30904044 10.1016/j.amjms.2019.01.004 PMC6863737

[4] Qi X, Liu Y, Wang J, et al. Clinical course and risk factors for mortality of COVID-19 patients with pre-existing cirrhosis: a multicentre cohort study [published online ahead of print. Gut. 2020 5 20;gutjnl-2020-321666. DOI:10.1136/gutjnl-2020-321666. PMC7815629 32434831

[5] National Health Commission of the People’s Republic of China. Chinese management guideline for COVID-19. (version 6.0). 2 19, 2020. http://www.nhc.gov.cn/yzygj/s7653p/202002/8334a8326dd94d329df351d7da8aefc2/files/b218cfeb1bc54639af227f922bf6b817

[6] European association for the study of the liver. EASL clinical practice guidelines for the management of patients with decompensated cirrhosis. J Hepatol. 2018;69(2):406–460. 29653741 10.1016/j.jhep.2018.03.024

[7] Chinese Society of Hepatology,Chinese Medical Association. Chinese guidelines on the management of liver cirrhosis. Zhonghua Gan Zang Bing Za Zhi. 2019;27(11):846–865. 31941240 10.3760/cma.j.issn.1007-3418.2019.11.008 PMC12770630

[8] Xiao Y, Pan H, She Q, et al. Prevention of SARS-CoV-2 infection in patients with decompensated cirrhosis. Lancet Gastroenterol Hepatol. 2020;5(6):528–539. 32197093 10.1016/S2468-1253(20)30080-7 PMC7270565

[9] Zhang C, Shi L, Wang FS. Liver injury in COVID-19: management and challenges. Lancet Gastroenterol Hepatol. 2020;5(5):428–430. 32145190 10.1016/S2468-1253(20)30057-1 PMC7129165

[10] Wang Y, Liu S, Liu H, et al. SARS-CoV-2 infection of the liver directly contributes to hepatic impairment in patients with COVID-19. J Hepatol. 2020;;73(4):807–816. 10.1016/j.jhep.2020.05.002 PMC7211738 32437830

[11] Bajaj JS, Khoruts A. Microbiota changes and intestinal microbiota transplantation in liver diseases and cirrhosis. J Hepatol. 2020;72(5):1003‐1027. 10.1016/j.jhep.2020.01.017 32004593

[12] Teltschik Z, Wiest R, Beisner J, et al. Intestinal bacterial translocation in rats with cirrhosis is related to compromised Paneth cell antimicrobial host defense. Hepatology. 2012;55(4):1154‐1163. 10.1002/hep.24789 22095436

[13] Guan WJ, Liang WH, Zhao Y, et al. Comorbidity and its impact on 1590 patients with COVID-19 in China: a nationwide analysis. Eur Respir J. 2020 2020 Published 514;55(5):2000547 . 32217650 10.1183/13993003.00547-2020 PMC7098485

[14] Newman KL, Johnson KM, Cornia PB, et al. Perioperative evaluation and management of patients with cirrhosis: risk assessment, surgical outcomes, and future directions [published online ahead of print. Clin Gastroenterol Hepatol. 2019;S1542-3565(19)30840–7. DOI:10.1016/j.cgh.2019.07.051. 731]. PMC6994232 31376494

[15] Zhou F, Yu T, Du R, et al. Clinical course and risk factors for mortality of adult inpatients with COVID-19 in Wuhan, China: a retrospective cohort study. Lancet. 2020;395(10229):1054‐1062. 10.1016/S0140-6736(20)30566-3 PMC7270627 32171076

[16] Wang D, Hu B, Hu C, et al. Clinical characteristics of 138 hospitalized patients with 2019 novel coronavirus-infected pneumonia in Wuhan, China. JAMA. 2020;323(11):1061‐1069. 10.1001/jama.2020.1585 PMC7042881 32031570

[17] Wang T, Chen R, Liu C, et al. Attention should be paid to venous thromboembolism prophylaxis in the management of COVID-19. Lancet Haematol. 2020;7(5):e362‐e363. 10.1016/S2352-3026(20)30109-5 PMC7158946 32278361

[18] Zhou P, Yang XL, Wang XG, et al. A pneumonia outbreak associated with a new coronavirus of probable bat origin. Nature. 2020;579(7798):270–273. 32015507 10.1038/s41586-020-2012-7 PMC7095418

[19] Sultan S, Altayar O, Siddique SM, et al. AGA Institute rapid review of the gi and liver manifestations of COVID-19, meta-analysis of international data, and recommendations for the consultative management of patients with COVID-19 [published online ahead of print. Gastroenterology. 2020 [55]];S0016-5085(20)30593-X. DOI:10.1053/j.gastro.2020.05.001 PMC7212965 32407808

[20] Xu L, Liu J, Lu M, et al. Liver injury during highly pathogenic human coronavirus infections. Liver Int. 2020;40(5):998–1004. 32170806 10.1111/liv.14435 PMC7228361

[21] Hu LL, Wang WJ, Zhu QJ, et al. Novel coronavirus pneumonia related liver injury: etiological analysis and treatment strategy. Zhonghua Gan Zang Bing Za Zhi. 2020;20,28(2):97–99. 10.3760/cma.j.issn.1007-3418.2020.02.001 PMC12769537 32075364

[22] Strnad P, Tacke F, Koch A, et al. Liver - guardian, modifier and target of sepsis. Nat Rev Gastroenterol Hepatol. 2017;14(1):55–66. 27924081 10.1038/nrgastro.2016.168

[23] Stengel S, Quickert S, Lutz P, et al. Peritoneal Level of CD206 associates with mortality and an inflammatory macrophage phenotype in patients with decompensated cirrhosis and spontaneous bacterial peritonitis. Gastroenterology. 2020 ;158(6):1745–1761. 10.1053/j.gastro.2020.01.029 31982413

[24] Pagliaro L. MELD: the end of Child-Pugh classification? J Hepatol. 2002;36(1):141‐142. 10.1016/s0168-8278(01)00302-6 11804679

[25] Peng Y, Qi X, Guo X Child-Pugh Versus MELD Score for the Assessment of Prognosis in Liver Cirrhosis: a Systematic Review and Meta-Analysis of Observational Studies. Medicine (Baltimore). 2016;95(8):e2877. 26937922 10.1097/MD.0000000000002877 PMC4779019

[26] Moon AW, Webb GJ, Aloman C, et al. High mortality rates for SARS-CoV-2 infection in patients with pre-existing chronic liver disease and cirrhosis. J Hepatol. 2020;20:S0168-8278(20)30305–6. 10.1016/j.jhep.2020.05.013 PMC7241346 32446714

[27] Barlow G, Nathwani D, The DP. CURB65 pneumonia severity score outperforms generic sepsis and early warning scores in predicting mortality in community-acquired pneumonia. Thorax. 2007;62(3):253–259. 16928720 10.1136/thx.2006.067371 PMC2117168

