# Supplementary material for: High Child-Pugh and CRUB65 scores predict mortality of decompensated cirrhosis patients with COVID-19: A 23-center, retrospective study
Source: Virulence. 2021 Apr 19;12(1):1199–208. doi: 10.1080/21505594.2021.1909894 (PMC8078510; doi:10.1080/21505594.2021.1909894)
Supplement: Supplemental Material [file KVIR_A_1909894_SM3857.docx]

**Supplementary Table 1 Case sources**

| **Hospital** | **COVID-19** | | |  |
| --- | --- | --- | --- | --- |
|  | **Decompensated Cirrhotic patients** | **Patients without liver Disease** | **Total** | |
| Wuhan Jinyintan Hospital | 11 | 44 | 2237 | |
| Renmin Hospital of Wuhan University | 6 | 24 | 1678 | |
| Tongren Hospital of Wuhan University | 5 | 20 | 1673 | |
| The Second Affiliated Hospital of Jianghan University | 5 | 20 | 1084 | |
| The Central Hospital of Xiaogan | 4 | 16 | 710 | |
| Hanyang Hospital Affiliated to Wuhan University of science and technology | 4 | 16 | 572 | |
| Third People's Hospital of Hubei Provincial | 4 | 16 | 584 | |
| Wuhan Hankou Hospital | 3 | 12 | 978 | |
| Tianyou Hospital, Wuhan University of Science and Technology | 3 | 12 | 786 | |
| Jingmen No.1 People’s Hospital | 3 | 12 | 425 | |
| Wuhan Ninth Hospital | 3 | 12 | 369 | |
| The Central Hospital of Wuhan | 2 | 8 | 858 | |
| Yichang Central People’s Hospital | 2 | 8 | 516 | |
| Xiang Yang No.1 People’s Hospital | 2 | 8 | 460 | |
| Union Hospital affiliated to Huazhong University of Science and Technology | 1 | 4 | 1568 | |
| Wuhan Fourth Hospital | 1 | 4 | 362 | |
| Chibi Central Hospital | 1 | 4 | 229 | |
| The First Affiliated Hospital of Nanchang University | 1 | 4 | 215 | |
| Tongji Xianning Hospital, Huazhong university of science and technology | 1 | 4 | 159 | |
| Central Theater General Hospital | 1 | 4 | 82 | |
| The First People’s Hospital of Tianmen | 1 | 4 | 67 | |
| Xiantao First People’s Hospital | 1 | 4 | 63 | |
| Tongcheng People's Hospital | 1 | 4 | 57 | |
| **Total** | **66** | **264** | **15732** | |
